# Parental attitudes and information needs in an adolescent HPV vaccination programme

**DOI:** 10.1038/sj.bjc.6604766

**Published:** 2008-11-04

**Authors:** R Stretch, S A Roberts, R McCann, D Baxter, G Chambers, H Kitchener, L Brabin

**Affiliations:** 1 Academic Unit of Obstetrics and Gynaecology, University of Manchester, Manchester M13 OJH, UK; 2 Health Methodology Research Group, University of Manchester, Manchester M13 9PT, UK; 3 Greater Manchester Health Protection Unit, Eccles M30 0NJ, UK; 4 Public Health Department, Stockport Primary Care Trust, Stockport SK4 1BS, UK; 5 Public Health Department, Bury Primary Care Trust, Bury M45 7TA, UK

**Keywords:** adolescent HPV vaccination, parents, schools, Cervarix, acceptability, information

## Abstract

We sent a questionnaire to 38% (1084) of 2817 parents whose daughters had been offered human papillomavirus vaccination and who had agreed to participate. Of these, 60% (651) returned a questionnaire. Responses suggested that fact sheets and parent information evenings confirmed, rather than changed, consent decisions. The views of active refusers on safety and efficacy may be difficult to change, lowering vaccine coverage.

In the United Kingdom, routine human papillomavirus (HPV) vaccination for 12–13-year-old girls to prevent cervical cancer has begun. The highest possible vaccine uptake is required to achieve maximum impact on future cancer incidence and to ensure cost effectiveness (Goldhaber-Fiebert *et al*, 2008). Two parental acceptability studies in the UK anticipated an uptake of about 80% (Brabin *et al*, 2006; Marlow *et al*, 2007a), which would be similar to the coverage achieved by the cervical screening programme. As those girls who are not vaccinated may include some who would not take advantage of future cervical screening, reducing non-acceptance is important (Jit *et al*, 2008).

A school-based programme increases the possibility of high coverage, but the acceptability of vaccinating adolescents against a sexually transmitted infection remains uncertain, and the general public is relatively uninformed, or even misinformed, about cervical cancer and its prevention (Friedman and Shepheard, 2007; Marlow *et al*, 2007b). We assessed vaccine acceptability in a feasibility study ahead of the national vaccine programme. Two primary care trusts (PCTs) in Greater Manchester that offered Cervarix (GlaxoSmithKline, Rixensart, Belgium) to girls attending 36 secondary schools achieved a 71% uptake of the first HPV vaccine dose (Brabin *et al*, 2008). Here we present the results of a parental questionnaire survey shortly after the second dose, which focussed on factors that had influenced the parents’ vaccine decision and included a small group of respondents who had declined vaccination.

## Materials and methods

The North Manchester NHS Research Ethics Committee approved the study. Cervarix was offered at 0, 1 and 6 months to 2817 girls aged 12–13 years between October 2007 and July 2008 (Brabin *et al*, 2008). In the covering letter, parents were informed that the funding source was GlaxoSmithKline and that Cervarix was one of two licensed vaccines; it explained that the vaccine for the future national immunisation programme and the policy for vaccinating older girls had not yet been determined. Parents received information about cervical cancer and the vaccine, a flier summarising the content of an educational film for girls (Vallely *et al*, 2008), details of parent evenings and a separate consent form for the follow-up research questionnaire. Information evenings provided an overview of HPV vaccines and the study aims; the educational film was shown, followed by a question and answer session facilitated either by school nurses or by a consultant in communicable diseases.

Primary care trusts forwarded to the research team the names and addresses of parents who had agreed to be sent questionnaires. The questionnaire asked about factors that may have influenced vaccine consent, including socio-demographic characteristics; the information sheet; parent information evenings; other information sources; concerns about vaccine safety and efficacy; and their child's wish for vaccination and sexual issues. Responses were mainly measured using a Likert scale appropriate to the question asked. Proportions were summarised according to whether consent was given (‘consenters’) or refused (‘refusers’), and Fisher's exact tests were used to assess the significance of differences between groups. An open question asked parents who had attended an information session to state whether, and how, this had influenced their vaccine decision. The responses were analysed semi-qualitatively.

## Results

In all, 38% (1084) of the 2853 eligible parents consented to be contacted and 60% (651) of these returned a questionnaire, including 605 consenters and 46 refusers (20% of the non-vaccinated group). There were no significant differences in the ages, ethnicity, religion or free school meal entitlement between consenters and refusers in either PCT. Compared with the general population, fewer questionnaires were returned by parents of children receiving free school meals (6 *vs* 13%) or non-white parents (7 *vs* 10%), and only 17 were non-Christian.

In total, 97% (628) of the parents had read the information sheet. Compared with consenters, refusers were less satisfied with the level of detail provided, were more likely to state that it did not answer their questions and were largely uninfluenced by its contents (*P*<0.001) (Table 1). Parents were least clear about the length of protection conferred and how the vaccine prevented cervical cell changes (Table 2). Refusers were more likely than consenters to remain unclear about the results of clinical trial data (16 *vs* 5%; *P*=0.01) and HPV types (14 *vs* 3%; *P*=0.004).

The 20% (128) of parents who attended an information evening comprised 32% (14) refusers and 19% (114) consenters (*P*=0.049). Of the 90% (115) who expressed their view on the evening, 26% (30) stated that it had no influence on their vaccine decision. Some parents valued the opportunity to talk to a health professional for ‘independent’ advice and to hear more detailed information, explained in a way they understood. They liked to hear the views of other parents, which introduced them to new issues, and found the discussions useful and enjoyable. Predominantly, parents used words such as ‘reassured’, ‘confirmation’ and ‘confidence’, although for refusers this generally signified confirmation that other parents shared their reservations.

In all, 33% (215) of the parents gained information on the vaccine from television, 24% (152) from newspapers, 18% (113) from the internet and 6% (100) from a healthcare provider. Friends and relations (14%), radio (13%) and magazines (6%) were less often cited. Only 14% (88) based their decision solely on the information provided by the vaccine programme. Refusers actively sought additional information more often than consenters, citing the internet or health professionals as sources (48 *vs* 27%, *P*=0.006).

More refusers than consenters had concerns about vaccine safety in general, side effects and booster doses (Table 3). Compatibility of the two available vaccines worried 57% (26) of refusers but only 16% (94) of consenters (*P*<0.001). Asked whether the HPV vaccine would encourage their child to become more sexually active, 77% (494) responded ‘not at all’, 19% (124) ‘not much’, 3% (21) ‘quite’ and 1% (3) were ‘very’ concerned. Refusers were more likely to be ‘very’ or ‘quite’ concerned (11% (5) *vs* 3% (19); *P*=0.021).

In addition, 97% (628) of parents reported discussing the vaccine with their daughters. Asked whether their daughters wished to be vaccinated, consenters and refusers, respectively, responded ‘Yes’ [83% (487) *vs* 19% (8)]; ‘No’ [4% (25) *vs* 50% (21)]; ‘It was not her decision’ [11% (63) *vs* 29% (12)]; or ‘Don’t Know’ [2% (11) *vs* 2% (1)] (*P*<0.001)]. They did not differ with regard to whether boys should be vaccinated. Altogether 52% (328) said ‘Yes’, 5% (33) said ‘No’, 32% (203) would ‘Leave it to the experts to decide’ and 11% (72) ‘Didn’t Know’.

## Discussion

Although parents who responded were not familiar with HPV vaccination, the information they received through PCTs only partly influenced their vaccine decision. They mainly sought reassurance about vaccine safety, but as the vaccine is new and phase 4 trials are ongoing doubts about its long-term safety cannot be fully answered. As long as safety remains an important issue, adolescent HPV vaccine coverage may, like the measles, mumps and rubella (MMR) vaccine (Smith *et al*, 2007), not reach the desired level, or that achieved for most infant vaccines. Of concern is the fact that 50% of refusers stated their daughters did not wish to be vaccinated because we do not know whether these girls will take advantage of cervical screening in future.

This is the first study to address parental acceptance of adolescent HPV vaccination within a vaccine programme. Inevitably, it is likely that those responding over-represent the more engaged, articulate parents with stronger views. A return rate of 60%, representing a quarter of the general population, is comparable to telephone surveys on HPV acceptability (Constantine and Jerman, 2007; Ogilvie *et al*, 2007) and higher than a Dutch postal survey (Lenselink *et al*, 2008). Parents who do not return questionnaires may also be less responsive to a vaccine invitation. A recent study of 14-year-old Belgian adolescents reported lower general vaccine coverage rates for children of single, divorced parents and larger families (⩾4 children) (Vandermeulen *et al*, 2008). The sample did include active refusers (7% of the sample compared with 8% of the population) who tend to be better educated and may hold strong beliefs, but we do not have any information about those who did not respond to the vaccine invitation, and more work is required to understand this group.

Dempsey *et al* (2006) reported no effect of written information on HPV vaccine acceptability. We further report that information evenings were attended by a minority of parents, with refusers most likely to attend, whose views were not substantially altered as a result. The literature on childhood vaccination shows that parents who believe in vaccination tend to comply with, rather than make, an informed decision. (Tickner *et al*, 2007). Worries about MMR have increased public scepticism; therefore, health professionals giving information to parents need to be well prepared with robust, up-to-date information on vaccine safety and other issues. Some refusers cited concerns about vaccine compatibility. This arose from a perception that a quadrivalent must be inherently ‘better’ than a bivalent vaccine, especially as other countries had already selected it. Misinterpretation of the licensing process led to parents waiting to see if the quadrivalent vaccine would be selected for the national programme, even though their daughters might not be eligible (i.e. if there were no catch-up programme). Tailored written information on safety issues could also be prepared, but parents may have taken a decision based on beliefs and attitudes that are difficult to modify.

## Conclusions

Despite some unease about the safety of HPV vaccination, most parents who responded wished to protect their daughters from cervical cancer and comply with vaccine recommendations. Although there is no evidence of bias, the responders represent a quarter of the population in two PCTs; hence, caution is needed in extrapolating the results to the general population. It remains uncertain whether HPV vaccination coverage will exceed cervical screening coverage. Parents may listen to health professionals, who should aim to raise the uptake by communicating the latest scientific data to refusers and dispelling misperceptions about the vaccine.

## Figures and Tables

**Table 1:** Respondent's view of the information sheet

		**Consenters**		**Refusers**		
	**Missing**	***n***	**%**	***n***	**%**	***P*-value^a^**
*Did you find the information sheet easy to read?*						
Yes		404	69	29	67	0.784
Mostly	26	158	27	12	28	
Partly		20	4	2	5	
						
*Did it give the level of detail you wanted?*						
Too little		65	11	20	47	<0.001
The right amount	29	511	88	23	53	
Too much		3	1	0	0	
						
*Did you find the information sheet answered your questions?*						
Yes		239	41	5	12	<0.001
Mostly	29	285	49	15	35	
Partly		52	9	17	39	
No		3	1	6	14	
						
*Did the information sheet influence your decision?*						
A lot		79	14	2	5	<0.001
Quite a lot	34	238	41	9	21	
A little		190	33	17	41	
Not at all		68	12	14	33	

a Fisher's exact test.

**Table 2:** Parental assessment of the clarity of the facts in the information sheet

		**Consenters**		***Refusers***		
**Facts**	**Missing**	***n***	**%**	***n***	**%**	***P*-value**
*HPV is the main cause of cervical cancer*						
Very clearly		285	49	22	50	1
Clearly	30	283	49	22	50	
Not clearly		9	2	0	0	
						
*Adolescents are at risk of HPV when they start having sex*						
Very clearly		284	49	27	62	0.24
Clearly	31	279	49	16	36	
Not clearly		13	2	1	2	
						
*Vaccine prevents HPV 16 and 18*						
Very clearly		258	45	20	46	0.004
Clearly	32	301	52	18	41	
Not clearly		16	3	6	14	
						
*Clinical trials show vaccine prevents persistent infection*						
Very clearly		214	37	16	37	0.010
Clearly	34	334	58	20	47	
Not clearly		26	5	7	16	
						
*The vaccine prevents changes to cervical cells*						
Very clearly		155	27	8	19	0.069
Clearly	34	354	62	25	58	
Not clearly		65	11	10	23	
						
*The vaccine provides protection for at least 5 years*						
Very clearly		165	29	13	30	0.32
Clearly	34	310	54	19	44	
Not clearly		99	17	11	26	
						
*Condoms do not offer 100% protection against HPV*						
Very clearly		218	38	15	37	0.97
Clearly	35	304	53	23	56	
Not clearly		53	9	3	7	
						
*Women will still need to go for cervical smears*						
Very clearly		265	46	17	43	0.81
Clearly	37	269	47	21	52	
Not clearly		40	7	2	5	
						
*The vaccine does not protect if you already have HPV 16/18*						
Very clearly		212	37	14	35	0.58
Clearly	42	301	53	24	60	
Not clearly		56	10	2	5	

**Table 3:** Efficacy and safety concerns affecting HPV vaccine acceptance among parents who consented or refused consent (numbers and percentage of respondents who had ‘a lot’ or ‘quite a lot’ of concerns)

			**Consenters**		**Refusers**		
		**Missing**	***n***	**%**	***n***	**%**	***P*-value^a^**
Influence of MMR^b^ debate	Yes	10	107	18	24	53	<0.001
Daughter may have a bad reaction	Yes	10	82	14	17	38	<0.001
Long-term side effects	Yes	10	111	19	23	51	<0.001
Need for a booster	Yes	11	60	10	18	40	<0.001
Uncertainty about compatibility with other HPV vaccines	Yes	9	94	16	26	57	<0.001

a Fisher's exact test.

b MMR=measles, mumps and rubella vaccination.
